# Shining light on the future of radiology: addressing medical students’ perceptions

**DOI:** 10.1007/s00330-025-11615-z

**Published:** 2025-04-17

**Authors:** Emily Hoffmann, Inka Ristow

**Affiliations:** 1 Clinic of Radiology, University of Münster, Münster, Germany; 2 Department of Diagnostic and Interventional Radiology and Nuclear Medicine, University Medical Center Hamburg-Eppendorf, Hamburg, Germany

The field of radiology is facing a paradox: while its clinical importance is widely recognized, many medical students are hesitant to pursue radiology as a career. The recent study by Seng et al [1] explores this issue by analyzing the perceptions of medical students regarding radiology as a specialty. Their findings provide crucial insights into the reasons why radiology remains an under-considered career choice and suggest potential strategies to improve its attractiveness among future physicians.

Radiology plays a central role across almost all medical disciplines; nevertheless, the proportion of teaching hours dedicated to this specialty within the medical curriculum is rather low compared to core disciplines such as internal medicine or surgery [2]. This disparity results in medical students receiving limited interaction with the specialty, thereby not accurately reflecting the diagnostic and practical demands of our profession. Conventional educational approaches, such as large-group lectures, focus primarily on imparting theoretical knowledge and do not adequately prepare students for the hands-on aspects of working as a radiologist. The need for curricular innovation emphasizes the importance of research efforts to understand medical students’ perceptions of radiology. By assessing their perspectives, educational reforms can more appropriately address the gap between traditional teaching methods and interactive, hands-on experiences that truly foster student interest.

In their article “Counting coins in the dark—Austrian, German and Swiss medical students’ perceptions of Radiology”, Seng et al surveyed 1184 medical students across Austria, Germany, and Switzerland to assess their attitudes towards radiology. The results reveal a striking contradiction: while 99% of students acknowledged the clinical relevance of radiology, a majority (64%) ruled out a career in the field. The primary deterrent was a perceived lack of patient interaction (cited by 62% of students), followed by the concern that artificial intelligence (AI) could replace the profession in the future (33%). On the other hand, salary prospects and work-life balance were seen as major advantages. Interestingly, 60% of respondents wished for greater exposure to radiology in their curriculum. Subgroup analyses indicated that prior exposure to radiology—through internships or research—significantly increased the students’ interest in the field.

The findings emphasize the need for targeted educational interventions to improve the image of radiology among medical students. Although the COVID-19 pandemic has significantly transformed medical teaching concepts in recent years, accelerating the adoption of digital learning tools and remote teaching strategies, these changes have also been accompanied by notable challenges, including reduced hands-on training, limited practical experience, particularly in interventional radiology, and a greater disparity in access to high-quality resources [3, 4]. These changes highlighted the need for a balanced integration of digital innovations with essential in-person training for comprehensive training of medical students in radiology.

Limited patient interaction remains a significant deterrent, but this perception could be mitigated by the integration of more interactive learning experiences, such as bedside radiology, interdisciplinary case discussions, virtual reality and augmented reality simulations [5, 6]. However, this requires students to be able to apply and evaluate these techniques in a reflective manner [7]. Enhancing exposure through simulation-based training or active participation in sonographic and interventional procedures could further bridge the gap between radiology and direct patient care [8].

Furthermore, identified concerns about AI highlight the need to clarify its current and future role in radiology. Rather than replacing radiologists, AI is set to enhance their capabilities by optimizing workflows and improving diagnostic accuracy. This allows radiologists to focus on their core tasks, which is especially important considering the increasing workload due to the growing complexity and volume of data to be analyzed. Given the impact AI is already having on healthcare and will continue to have in the future, it is important that sufficient attention is dedicated to this continuously growing field in the medical curriculum. Specific AI and technology modules in medical education could help alleviate misconceptions and unfounded fears. For example, in the German National Competence Catalog for Medicine (NKLM), it is stated that, as part of the core curriculum, medical students should be familiar with current developments in digitalization in medicine and the field of artificial intelligence, including machine learning methods, personalized medicine, clinical decision support systems, and digital image processing [9]. In addition, users should be able to name the chances and limitations of these techniques. At the present time, however, it must be noted that neither those already practicing medicine nor the generation currently studying medicine have been adequately prepared for the integration of AI into routine medical practice, although they do find themselves in an increasingly technology-dominated working environment.

Finally, motivating students by highlighting how they can contribute to and shape the future of AI in medicine will encourage a more proactive engagement with this transformative field. This engagement can ensure that the next generation of radiologists is well-prepared to leverage AI to improve patient outcomes.

This study serves as a wake-up call for radiology departments and academic institutions. To ensure a steady influx of radiologists in the coming years, proactive steps must be taken to counteract stereotypes and highlight the field’s diversity, intellectual stimulation, and patient-centered approach [10].

Future efforts should focus on longitudinal studies to evaluate the impact of curricular adaptations on students’ career choices. Moreover, collaborations between radiology societies, universities, and student organizations could help design targeted outreach programs to sustain interest in the field.

To conclude, radiology is a critical component in modern personalized medicine, but its future workforce faces challenges due to persistent misconceptions. The study by Seng et al underscores the urgent need for curricular reforms and innovative recruitment strategies. By highlighting the dynamic and constantly evolving nature of radiology, we should encourage future generations of physicians to pursue this multifaceted and innovative specialty.
